# Evolution of a key trait greatly affects underground community assembly process through habitat adaptation in earthworms

**DOI:** 10.1002/ece3.3777

**Published:** 2018-01-08

**Authors:** Hiroshi Ikeda, Kayoko Fukumori, Etsuko Shoda‐Kagaya, Masamichi Takahashi, Masamichi T. Ito, Yoshimi Sakai, Kazuma Matsumoto

**Affiliations:** ^1^ Faculty of Agriculture and Life Science Hirosaki University Hirosaki Japan; ^2^ National Institute of Advanced Industrial Science and Technology Tsukuba Japan; ^3^ Forestry and Forest Products Research Institute Tsukuba Japan; ^4^ Faculty of Economics and Management Surugadai University Hanno Japan; ^5^ Kyushu Research Center Forestry and Forest Products Research Institute Kumamoto Japan; ^6^ Association of International Research Initiatives for Environmental Studies Taito‐ku Japan

**Keywords:** earthworm, geographic distance, habitat environment, multilocus species delimitation, niche conservatism

## Abstract

Underground community assemblies have not been studied well compared with aboveground communities, despite their importance for our understanding of whole ecosystems. To investigate underground community assembly over evolutionary timescales, we examined terrestrial earthworm communities (Oligochaeta: Haplotaxida) in conserved mountainous primary forests in Japan as a model system. We collected 553 earthworms mostly from two dominant families, the Megascolecidae and the Lumbricidae, from 12 sites. We constructed a molecular taxonomic unit tree based on the analysis of three genes to examine the effects of a biogeographic factor (dispersal ability) and an evolutionary factor (habitat adaptation) on the earthworm community assembly process. The phylogenetic distance of the earthworm communities among sites was positively correlated with geographic distance when intraspecific variation was included, indicating that the divergence within species was affected by biogeographic factors. The community assembly process in the Megascolecidae has also been affected by environmental conditions in relation to an evolutionary relationship between habitat environment and intestinal cecum type, a trait closely related to habitat depth and diet, whereas that in the Lumbricidae has not been affected as such. Intestinal cecum type showed a pattern of niche conservatism in the Megascolecidae lineage. Our results suggest that investigating the evolution of a key trait related to life history can lead to the clear description of community assembly process over a long timescale and that the community assembly process can differ greatly among related lineages even though they live sympatrically.

## 1 INTRODUCTION

In terrestrial ecosystems, too little research has been conducted on the detritus food chain compared with the grazing food chain, most constituents of which live underground (Decaëns, 2010). Aboveground community assemblages and their functions are closely related to underground community assemblages (Toju, Guimarães, Olesen, & Thompson, 2015; Wardle et al., 2004). Thus, clarifying the factors driving the underground community assembly process is important for our understanding of whole ecosystems.

The community assemblage in an area forms by the expansion of the distribution range of each component organism over an evolutionary timescale. Two main factors explain why a species lives in a particular place (Webb, Ackerly, McPeek, & Donoghue, 2002): biogeographic factors control whether the species is able to arrive at that place via dispersal, and evolutionary factors control whether the ancestor of the species had evolved the traits necessary to live in that particular environment. Related species tend to share similar ecological traits that are adaptive in similar environments (a phenomenon known as ecological niche conservatism), although related species may also evolve different habitat preferences as a consequence of adaptive radiation (Emerson & Gillespie, 2008). Thus, if habitat adaptation is conserved through the phylogeny, and no competitive exclusion occurs, community assemblages at sites with similar environments tend to be composed of phylogenetically closely related species (Vamosi & Vamosi, 2007; Webb et al., 2002).

Studies of the community assembly process over evolutionary timescales have been conducted on aboveground plants and insects and in aquatic ecosystems, but few such studies have investigated organisms living underground. The underground environment is very different from those aboveground, in showing less annual and seasonal variability. In addition, soil animals have a poor dispersal ability and consequently show high genetic divergence among areas (Chang, Lin, & Chen, 2008; Minamiya, Yokoyama, & Fukuda, 2009). Therefore, the process of forming animal community assemblages underground is expected to differ greatly from that aboveground. Although several community phylogenetic studies have investigated soil animals such as ants (Smith, Hallwachs, & Janzen, 2014) and earthworms (Decaëns et al., 2016), these studies did not consider the evolutionary ecology of habitat adaptation, which can greatly affect community assembly.

In this study, we address the effects of biogeographic and evolutionary factors on the community assembly process of underground animals by using terrestrial earthworms (Oligochaeta: Haplotaxida) as a model system. Earthworms are appropriate representatives of soil animals, as they make large contributions to the decomposition of litter and soil organic matter (Darwin, 1881; Edwards & Lofty, 1977) and behave as ecosystem engineers that create and destroy habitat for other organisms by modifying the habitat where they live (Lavelle et al., 2016). We examined their dispersal ability as a biogeographic factor and their habitat adaptation as an evolutionary factor. Numerous invasive earthworms are known worldwide (Blakemore, Ito, Kaneko, et al. 2006; Hendrix et al., 2008), so we set our 12 study sites in conserved mixed coniferous–broadleaved or beech forests at relatively high elevations (650–1,920 m), which we expected to contain undisturbed soil animal communities without invasive earthworms.

**Figure 1:**
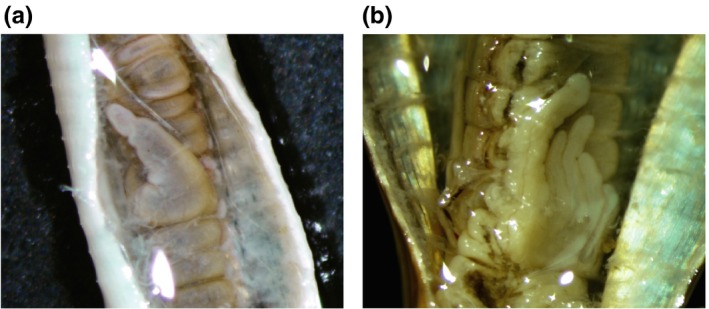
Intestinal ceca of megascolecid earthworms: (a) simple type; (b) manicate type

The Megascolecidae and the Lumbricidae are the dominant earthworm families in Japan. Megascolecid earthworms dominate with high diversity across Asia (Blakemore, Chang, Chuang, et al, 2006; Blakemore, Ito, Kaneko, et al, 2006; Ishizuka, 2001; Ishizuka & Minagoshi, 2014), whereas lumbricid earthworms show low diversity, although this family is more diverse in western Eurasia (Blakemore, 2003; Blakemore, Chang, Chuang, et al, 2006; Ishizuka & Minagoshi, 2014; Lehmitz et al., 2014; Perel, 1977). Most megascolecid species distributed in Japan belong to the genus *Pheretima s. lat*. (Ishizuka, 2001; Ishizuka & Minagoshi, 2014). The ecological niches of megascolecid earthworms are related to their intestinal cecum type; Japanese native megascolecid earthworms have a pair of intestinal ceca connected with the intestinal tract (Ishizuka, 2001; Ishizuka & Minagoshi, 2014). The shape of the intestinal cecum is related to the digestive enzyme that it produces (Nozaki, Ito, Miura, & Miura, 2013), which in turn is related to ecological factors such as habitat depth and diet (Ishizuka, 2001; Ishizuka & Minagoshi, 2014; Uchida et al., 2004). Those species with the simple (Figure 1a) and serrate types of ceca live in the soil layer and feed on decomposed organic matter; those with serrate ceca live in the deep soil layer and are seldom collected from the topsoil layer. Species with the manicate type (Figure 1b) live in the litter layer and feed on less decomposed organic matter. Owing to the evolution of different cecum types among members of the Megascolecidae, we expect that the community assembly process of megascolecid earthworms in Asia is more strongly affected by evolutionary factors than by biogeographic factors. We hypothesize that the evolution of intestinal cecum type in the Megascolecidae has led to an evolutionary change in habitat environment and thus affects community assembly of this family.

## 2 MATERIALS AND METHODS

### 2.1 Study sites and methods

**Figure 2:**
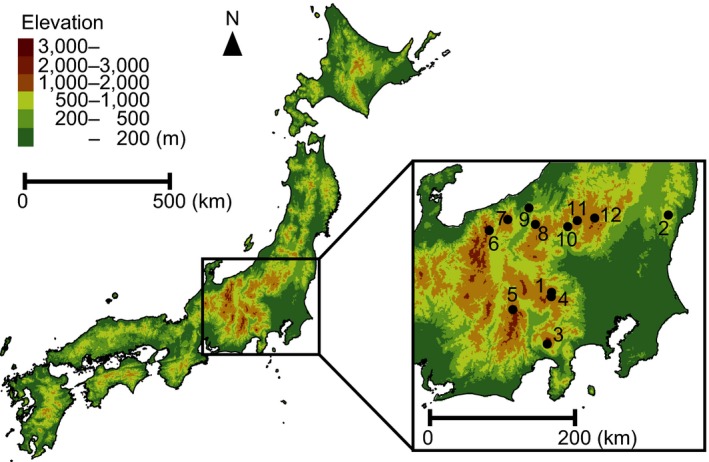
Map showing the location of the 12 study sites in central Japan. The map is constructed from 50 m‐mesh altitude data (Geospatial information authority of Japan 2001)

We conducted our study at 12 sites (20 m × 20 m) in primary mixed coniferous–broadleaved forests or beech forests in mountainous areas in the central part of mainland Japan (Figure 2, Appendix S1). We collected earthworms in early summer and in early autumn (sites 1 and 2: 4 and 6 July, 24 and 26 August 2010; sites 3–12: 1 to 14 July, 30 August to 8 September 2012). At each site, we used five quadrats (50 cm × 50 cm) for litter‐layer and soil‐layer sampling each time. We set these quadrats to be uniformly distributed at each site. Earthworms were hand‐collected from the litter layer and the 0‐ to 15‐cm soil layer in each quadrat and were preserved in 99% ethanol. This sampling depth (0–15 cm) is sufficient to collect most earthworms (Kaneda, Nozaki, & Fujii, 2012).

### 2.2 Morphology of earthworms

We identified earthworm specimens to the family level from their morphology. Because most earthworm species in Japanese primary forests have not been described, no morphological identification keys of earthworms at the species level have been established (Ikeda et al., 2012; Ishizuka, 2001; Minamiya et al., 2009). In addition, most collected samples were juveniles, which can be identified only to the family level from morphology. Thus, we used molecular operational taxonomic units (MOTUs) in this study (see “Phylogenetic analysis” section for details). We also examined the intestinal cecum of all intact megascolecid earthworms.

### 2.3 Environmental data

To classify the habitat environment of each earthworm, we measured environmental parameters reported to affect the earthworm community assembly process (Boettcher & Kalisz, 1991; Dotson & Kalisz, 1989; Lavelle, 1983; Spiers, Gagnon, Nason, Packee, & Lousier, 1986) at or near each quadrat where earthworms were collected. In the litter layer, litter depth in summer and autumn, dry weight, carbon content, and C:N ratio were measured. In the soil layer, depth of the A_1_ layer, bulk density, water content, pH, carbon content, and C:N ratio were measured (Appendix S1). In early summer and early autumn, we measured the depth of the leaf litter layer at five points. In early summer, we measured the depth of the A_1_ layer at five points and collected leaf litter samples (litter and fragmented decomposing litter) within a 25 cm × 25 cm area on the forest floor and surface soil (0–4 cm layer) using a 400‐ml cylindrical sampler.

We measured the wet weight of collected litter samples and the dry weight after oven‐drying them at 70°C for more than 3 days. Large branches, which are not consumed by earthworms, were removed. Dried litter samples were ground, and carbon and nitrogen contents were measured with a Sumigraph NC‐22F CN analyzer (Sumika Chemical Analysis Service, Ltd., Tokyo, Japan). The measured data were calibrated against an acetanilide standard.

Soil samples were oven‐dried at 70°C for more than 3 days and then passed through a sieve with 2‐mm‐diameter openings to remove gravel, stones, and roots. The gravel and stones were oven‐dried at 105°C overnight, and roots were oven‐dried at 105°C for a day to measure their dry weight. To calculate water content, we oven‐dried 3 g of each soil sample at 105°C overnight. We calculated the soil bulk density per 400 ml from these data. We put 10 g of oven‐dried soil into 25 g of ion‐exchanged water to measure soil pH on a portable pH meter (Horiba, Kyoto, Japan). Dried soil was ground, and carbon and nitrogen contents were measured with a Sumigraph NC‐22F CN analyzer.

We conducted principal component analysis for the average value of each environmental parameter at each site in R v. 3.2.4 software with standardization by the “prcomp” command (R Core Team, 2016). We considered the difference in PC scores between sites to represent the difference in habitat environment for earthworms between sites.

### 2.4 DNA sequencing

Total genomic DNA of earthworms was extracted using PrepMan Ultra Reagent (Applied Biosystems, Foster City, CA, USA). The following primers were used for PCR amplification and direct sequencing: mitochondrial *COI* gene: LCO1490 (forward), 5′‐GGT CAA CAA ATC ATA AAG ATA TTG G‐3′ (Folmer, Black, Hoeh, Lutz, & Virjenhoek, 1994), COI2198E (reverse), 5′‐TAW ACT TCW GGG TGW CCR AAR AAT CA‐3′ (modified from Bely & Wray, 2004; Folmer et al., 1994); mitochondrial *16S* gene: 16SF2 (forward), 5′‐CGA CTG TTT AAC AAA AAC ATT GC‐3′ (Pérez‐Losada, Ricoy, Marshall, & Domínguez, 2009), 16SR2 (reverse), 5′‐GTT TAA ACC TGT GGC ACT ATT C‐3′ (Pérez‐Losada et al., 2009); and nuclear *H3* gene: H3F (forward), 5′‐ATG GCT CGT ACC AAG CAG ACV GC‐3′ (Colgan et al., 1998), H3R (reverse), 5′‐ATA TCC TTR GGC ATR ATR GTG AC‐3′ (Colgan et al., 1998), H3R4 (reverse), 5′‐TGG GCA TGA TGG TGA CGC GCT‐3′ (this study). Purified PCR products were used in a dye terminator cycle‐sequencing reaction using these primers and a BigDye Terminator v. 3.1 Cycle Sequencing Kit (Applied Biosystems). The products were electrophoresed in an ABI 3130XL sequencer (Applied Biosystems). We obtained at least one gene region from all specimens. Sequence data are deposited in GenBank (GenBank accession numbers: LC199973–LC200394). We used MAFFT v. 7.273 software (qinsi method) for the *16S* alignment (Katoh & Standley, 2013). The alignments were inspected by eye for obvious misalignments. Haplotypes were detected using the “pgelimdupseq” command in Phylogears2 v. 2.0 software (Tanabe, 2008), and individuals with the same haplotypes were excluded from phylogenetic analyses.

### 2.5 Phylogenetic analysis

We used 876 bp of the *16S* gene, 637 bp of the *COI* gene, and 282 bp of the *H3* gene for phylogenetic analysis. None of the haplotypes including the combined sequences of the three genes were shared among sites. The optimum substitution models of each data set for phylogenetic analysis were estimated in Kakusan4 software (Tanabe, 2011), based on the Bayesian information criterion (BIC). Bayesian analysis was performed in BEAST v. 1.8.4 software (Drummond, Suchard, Xie, & Rambaut, 2012) with substitution models selected according to the BIC4 goodness‐of‐fit measure (*COI*: GTR+G; *16S*: GTR+G; *H3*: HKY+G). The *COI* sequences were partitioned by codon positions without unlinking parameters among codon positions. Enchytraeidae and Tubificidae samples were used as outgroups (GenBank accession numbers LC199985, LC199999, LC200017–LC200019, LC200023, LC200054, LC200069, LC200098, LC200152, LC200153, LC200156, LC200161, LC200175, LC200185, LC200203, LC200205, LC200232, LC200240, LC200242, LC200272, LC200325, LC200326, LC200329, LC200335, LC200349, LC200363, LC200385, LC228479–LC228482). First, we constructed a haplotype tree with all haplotypes to include the divergence within species. We used the log‐normal relaxed clock model for the analysis. The estimated clock rate for earthworms (Chang et al., 2008) was used as the prior for the clock rate of *COI* (initial value = 0.024). We performed three Markov chain Monte Carlo runs for 300 million generations, with trees sampled every 10,000 generations; the first 25,000 trees were discarded as burn‐in. We combined the remaining trees from three runs and used them for constructing a tree.

The node ages at the divergence points of the Megascolecidae + Lumbricidae and the Moniligastridae were used for setting the node age prior and for calibrating divergence times in the BEAST analysis. We tentatively calibrated the divergence point using a normal distribution (mean = 200 Mya, SD = 15.0), as the divergence of the Lumbricina is thought to be related to the breakup of Pangaea (Bouché, 1983; Domínguez et al., 2015; James & Davidson, 2012).

We split potential species (MOTUs) from the phylogenetic trees that include the divergence within species (haplotype tree), because most earthworm species have not been described in Japanese primary forests, and thus morphological identification keys have not been established (Ikeda et al., 2012; Ishizuka, 2001; Minamiya et al., 2009). We used a multilocus species delimitation method implemented in the program “tr2” (Fujisawa, Aswad, & Barraclough, 2016). The haplotype tree was used as a guide tree for analysis. We also constructed each gene tree for analysis in BEAST v. 1.8.4 software (Drummond et al., 2012). We used the same substitution models and settings for calibration that were used for the haplotype tree. We performed a Markov chain Monte Carlo run for 200 million generations, with sampling every 10,000 generations for each gene. The first 10,000 trees were discarded as burn‐in. We used the remaining trees for constructing each gene tree. The separated potential species were considered as species in the analysis to construct the MOTU tree.

We constructed the MOTU tree, in which the divergence among haplotypes within species was considered as the variation within species, in the program *BEAST (Heled & Drummond, 2010) in BEAST v. 1.8.4 software (Drummond et al., 2012). We used the same substitution models and settings for calibration that were used for the haplotype tree except for the random local clock model. We performed three Markov chain Monte Carlo runs for 400 million generations, with trees sampled every 10,000 generations, and the first 35,000 trees were discarded as burn‐in. We combined the remaining trees from the other three runs and used them for constructing trees. This MOTU tree was used for calculating the weighted Unifrac distance described below. We calculated rarefaction curves of MOTUs in EstimateS v. 9 software (Colwell, 2013) to compare species richness among families.

### 2.6 Testing phylogenetic distance of community assemblages among sites

**Figure 3:**
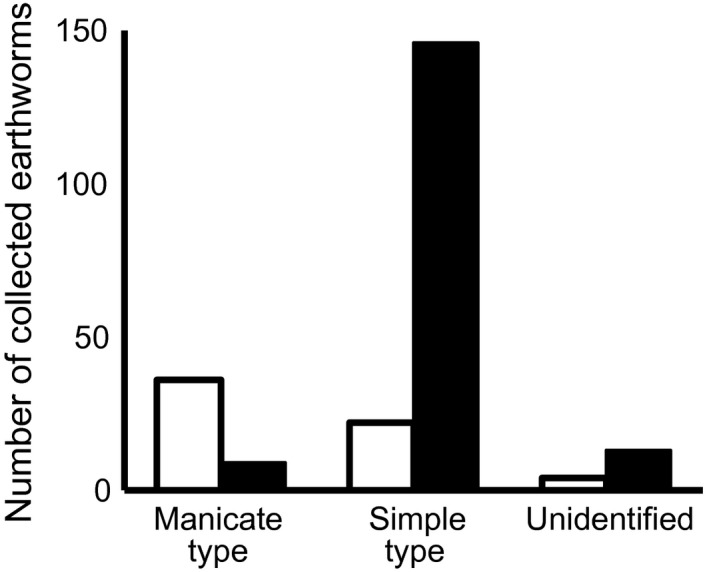
Numbers of earthworms collected from litter layer (□) and soil layer (■) according to intestinal cecum type

**Figure 4:**
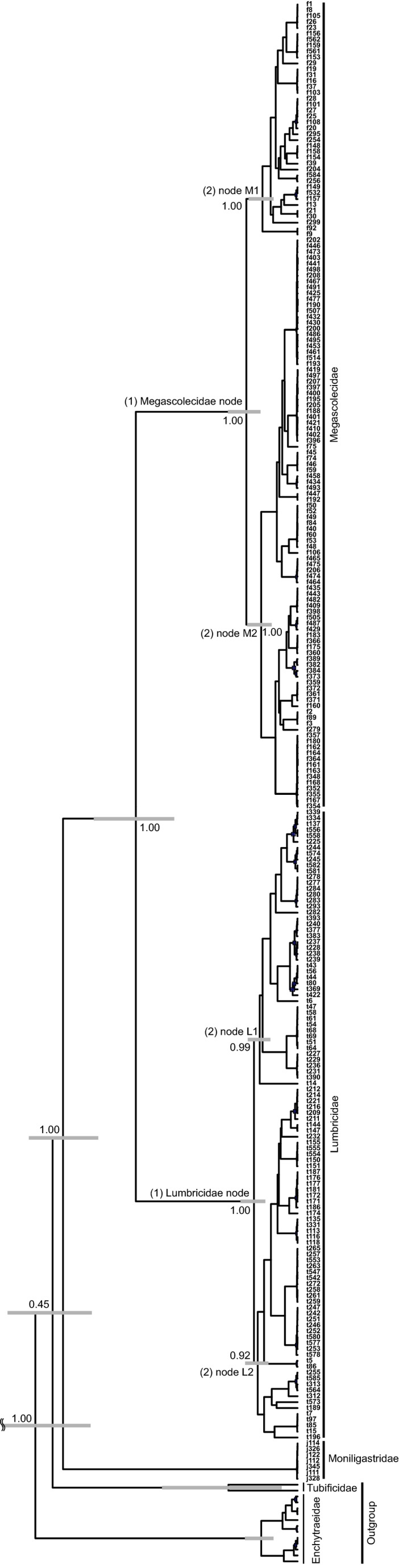
Haplotype tree with posterior probabilities shown for major in‐group nodes. Gray bars are 95% highest probability density intervals for the ages of major in‐group nodes. Three time points are also shown: (1) Megascolecidae node and Lumbricidae node; and (2) nodes within the Megascolecidae and within the Lumbricidae

To examine the phylogenetic distance of community assemblages among sites, we calculated weighted Unifrac distance using the MOTU tree by using the “unifrac” command in the phyloseq package (McMurdie & Holmes, 2013) in R. Unifrac distance is an index of the phylogenetic difference between community assemblages (Hamady, Lozupone, & Knight, 2010). When these communities are composed of the same or closely related species, the Unifrac distance between two communities is low. Therefore, if the community assembly process has been affected by biogeographic factors, a positive correlation between geographic distance and Unifrac distance is expected. On the other hand, if the community assembly process has been affected by evolutionary factors with niche conservatism, a positive correlation between environmental differences (PC1 and PC2 scores) and Unifrac distance is expected. We tested the positive correlation between weighted Unifrac distance and geographic distance or environmental differences (PC1 and PC2 scores). To remove the correlation between geographic distance and environmental differences (geographic distance and PC1 score: *p *=* *.042; geographic distance and PC2 score: *p *=* *.152 by Spearman's rank correlation test), we conducted a one‐tailed partial Mantel test with Spearman's rank correlation coefficient by using the “mantel” command with 1 million permutations in the “ecodist” package in R. Because the strength of the effect of each factor can vary over evolutionary timescales, we investigated the correlation at the following time points: (1) Megascolecidae node and Lumbricidae node; and (2) nodes within the Megascolecidae (see Figure 3). We also conducted the same analysis using the haplotype tree including the nodes within the Lumbricidae (see Figure 4).

### 2.7 Ancestral state reconstruction

To examine the evolutionary relationship between habitat environment and intestinal cecum type and the niche conservatism in these traits in the Megascolecidae, we reconstructed the ancestral states for habitat environments of megascolecid earthworms (PC1 and PC2 scores) by using parsimony ancestral state reconstruction with the squared change assumption in Mesquite v. 3.11 (Maddison & Maddison, 2016). PC1 and PC2 scores at the site where each earthworm sample was collected were set as the habitat environments for that sample. PC1 and PC2 scores of the species collected at more than one site were weighted by the number of samples collected at each site. Ancestral state reconstruction was conducted for the megascolecid MOTU tree. The ancestral states for intestinal cecum types of megascolecid earthworms were also reconstructed by parsimony ancestral state reconstruction in Mesquite v. 3.11 (Maddison & Maddison, 2016).

To examine the difference in habitat environments between the simple cecum type lineage and the manicate cecum type lineage, we used the phylogenetic generalized least squares method to control for phylogenetic signal by using the “pgls” command in the caper package (Orme et al., 2013) in R. We tested the correlation between the evolutionary change of intestinal cecum type and that of habitat environment using the MOTU tree. We compared AIC scores between the model with the evolutionary change in cecum type and the null model.

## 3 RESULTS

We collected 553 earthworms (230 Megascolecidae, 282 Lumbricidae, 41 Moniligastridae) in total from the litter and topsoil layers (Appendix S2) at the 12 sites (Figure 2, Appendix S1). Significantly more megascolecid earthworms with manicate ceca than with simple ceca were collected from the litter layer (Fisher's exact test, *p *<* *.001; Figure 3).

**Figure 5:**
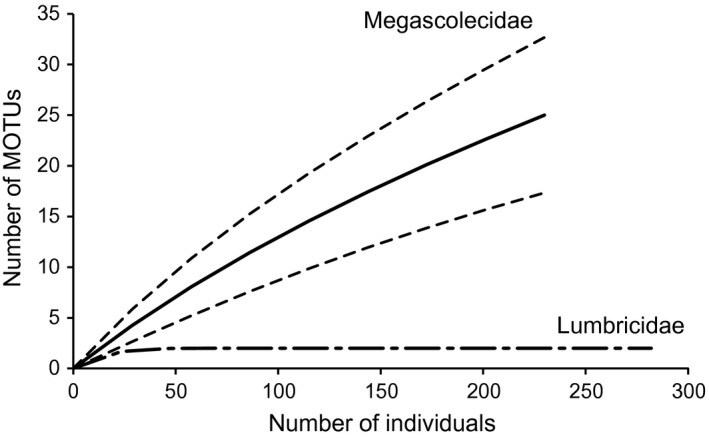
Individual‐based species rarefaction curves for Megascolecidae and Lumbricidae. Dashed lines represent 95% confidence intervals

**Figure 6:**
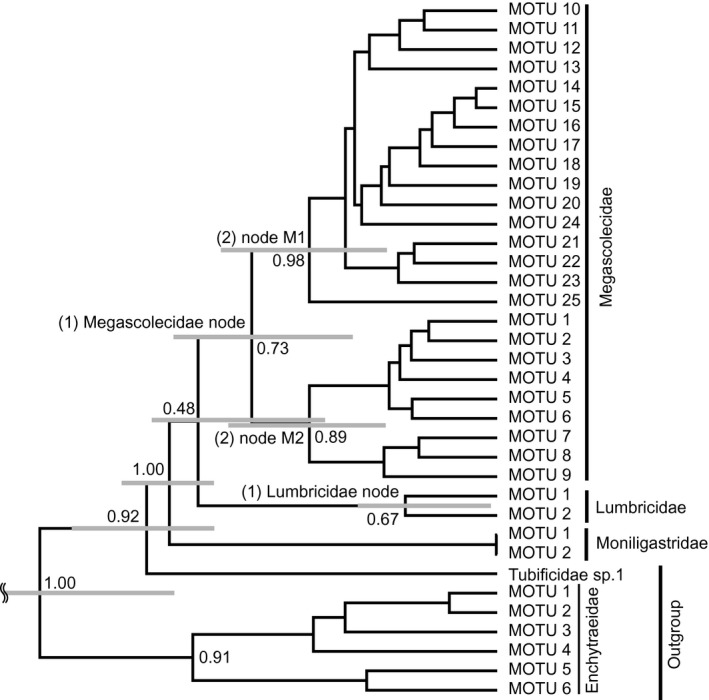
Molecular operational taxonomic unit (MOTU) tree with posterior probabilities shown for major in‐group nodes. Gray bars are 95% highest probability density intervals for the ages of major in‐group nodes. Particular time points are also shown: (1) Megascolecidae node and Lumbricidae node; and (2) nodes within the Megascolecidae

We estimated the number of MOTUs in each family as 25 in the Megascolecidae, 2 in the Lumbricidae, and 2 in the Moniligastridae from 251 haplotypes (137 in the Megascolecidae, 107 in the Lumbricidae, and 7 in the Moniligastridae). Rarefaction curves show that the Megascolecidae contain many more MOTUs than the Lumbricidae (Figure 5). We constructed the MOTU tree with these potential species (Figure 6).

The contribution of the first principal component (PC1) of the principal component analysis using environmental factors was 40.1%. PC1 was highly positively correlated with soil water content, proportion of carbon in soil, and depth of the A_1_ layer, and it was highly negatively correlated with soil bulk density (Table 1, Appendix S1). The contribution of PC2 was 25.0%. PC2 was highly positively correlated with C:N ratio in litter and soil, and it was highly negatively correlated with the proportion of carbon in litter and litter depth (Table 1, Appendix S1).

**Table 1:** PC1 and PC2 scores for each environmental factor

	PC1	PC2
Environmental factors for litter layer		
Litter depth in summer	0.328	−0.347
Litter depth in autumn	0.288	−0.347
Dry weight	0.165	−0.003
Carbon content	0.138	−0.370
C:N ratio	−0.222	0.452
Environmental factors for soil layer		
Depth of A1 layer	0.336	−0.105
Bulk density	−0.371	−0.319
Water content	0.430	0.094
pH	−0.270	−0.248
Carbon content	0.408	0.222
C:N ratio	0.197	0.431

**Figure 7:**
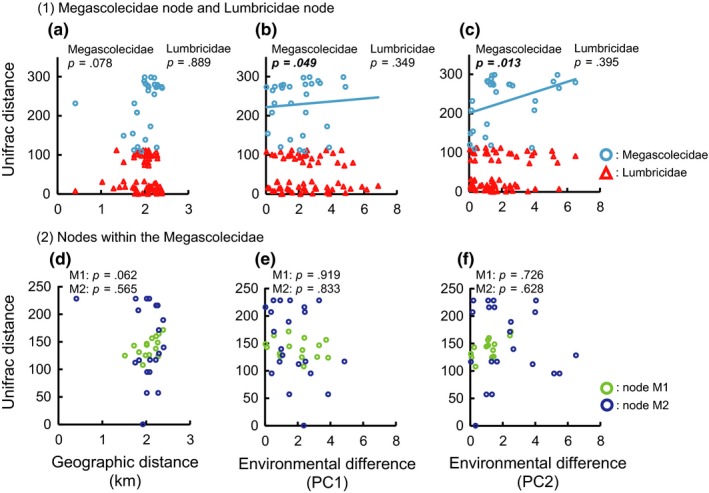
(1) Relationships of weighted Unifrac distance with (a) geographic distance and (b, c) environmental differences (PC1 and PC2, respectively) at Megascolecidae node and Lumbricidae node (*n *=* *28 for Megascolecidae, *n *=* *66 for Lumbricidae; see Fig. 6 for node points). (2) Relationships of weighted Unifrac distance with (d) geographic distance and (e, f) environmental differences (PC1 and PC2, respectively) at two nodes within the Megascolecidae (M1, *n *=* *15; M2, *n *= 21). These results are based on the MOTU tree. Statistical results of Spearman's rank correlation tests are also shown (see Table 2 for details). Significant *p* values are in bold italic type. Solid lines show the regression lines for significant relationships

**Figure 8:**
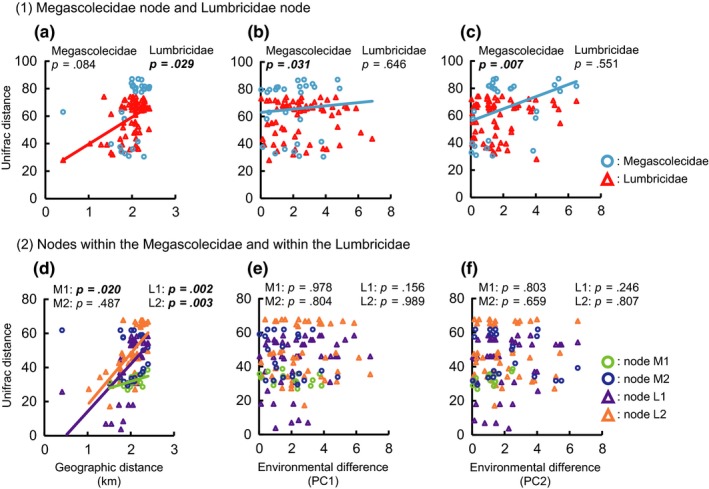
Based on the haplotype tree, (1) relationships of weighted Unifrac distance with (a) geographic distance and (b, c) environmental differences (PC1 and PC2, respectively) at Megascolecidae node and Lumbricidae node (*n* = 28 for the Megascolecidae, *n* = 66 for the Lumbricidae; see Fig. 4 for node points). (2) Relationships of weighted Unifrac distance with (d) geographic distance and (e, f) environmental differences (PC1 and PC2, respectively) at two nodes within the Megascolecidae (M1, *n* = 15; M2, *n* = 21) and two nodes within the Lumbricidae (L1, *n* = 45; L2, *n* = 45). Statistical results of Spearman's rank correlation tests are also shown (see Table 3 for details). Significant P values are in bold italic type. Solid lines show the regression lines for significant relationships

Our results showed that Unifrac distance was positively correlated with geographic distance for Lumbricidae lineages and for one Megascolecidae lineage in only the haplotype tree (Figures 7a,d and 8a,d; Tables 2 and 3). In contrast, it was positively correlated with PC1 and PC2 scores only at the Megascolecidae node in the MOTU tree and the haplotype tree (Figures 7b, c, e, f and 8b,c,e,f; Tables 2 and 3).

**Table 2:** Statistical results of Spearman's rank correlation tests of weighted Unifrac distance with geographic distance and environmental differences (PC1 and PC2)

	*n*	Geographic distance		Environmental difference			
				PC1		PC2	
		Mantel r	P value	Mantel r	P value	Mantel r	P value
(1) Megascolecidae node	28	0.25	.078	0.34	***.049***	0.41	***.013***
(1) Lumbricidae node	66	−0.17	.889	0.03	***.***349	0.02	.395
(2) Node M1 in Megascolecidae	15	0.49	.062	−0.38	***.***919	−0.18	.726
(2) Node M2 in Megascolecidae	21	−0.09	.565	−0.29	***.***833	−0.14	.628

Weighted Unifrac distances were based on the MOTU tree.

Significant *p* values are in bold italic font.

**Table 3:** Statistical results of Spearman's rank correlation tests of weighted Unifrac distance with geographic distance and environmental differences (PC1 and PC2)

	*n*	Geographic distance		Environmental difference			
		Mantel r	P value	PC1		PC2	
				Mantel r	P value	Mantel r	P value
(1) Megascolecidae node	28	0.25	.084	0.38	***.031***	0.46	***.007***
(1) Lumbricidae node	66	0.32	***.029***	−0.07	.646	−0.03	.551
(2) Node M1 in Megascolecidae	15	0.54	***.020***	−0.50	.978	−0.27	.803
(2) Node M2 in Megascolecidae	21	−0.04	.487	−0.24	.804	−0.15	.659
(2) Node L1 in Lumbricidae	45	0.60	***.002***	0.21	.156	0.09	.246
(2) Node L2 in Lumbricidae	45	0.66	***.003***	−0.33	.989	−0.20	.807

Weighted Unifrac distances were based on the haplotype tree. Significant *p* values are in bold italic font.

**Figure 9:**
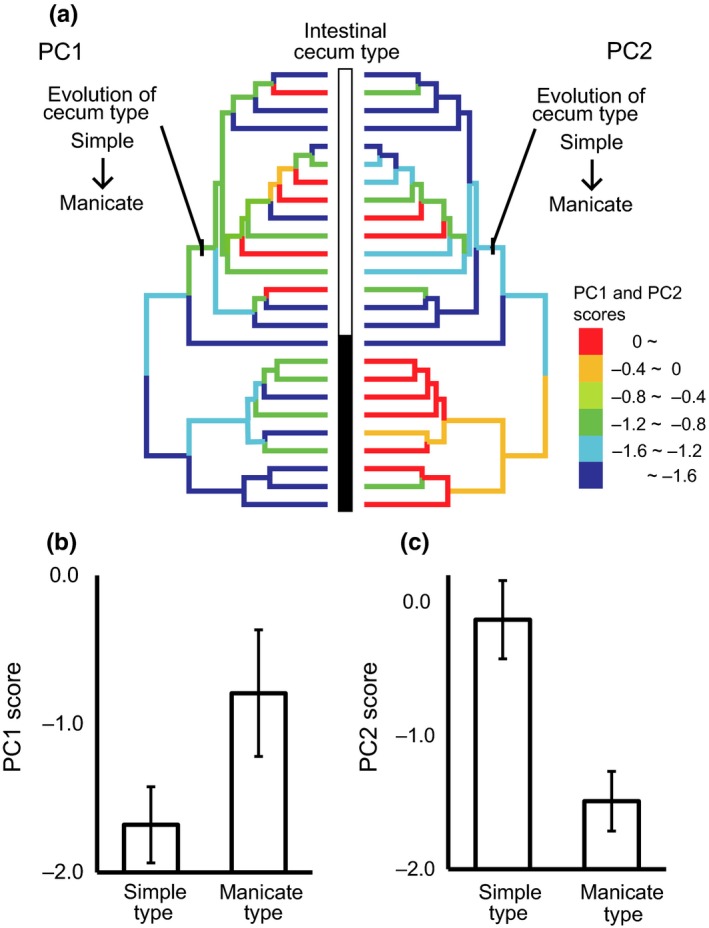
Ancestral state reconstruction of habitat environments and intestinal ceca and the difference in habitat environments between megascolecid earthworms with different ceca. (a) Ancestral state reconstruction for habitat environments (left, PC1 score; right, PC2 score) in the Megascolecidae. Intestinal cecum type for extant species (□ manicate type; ■ simple type) and the evolution of intestinal cecum type are also shown. The color on the phylogenetic tree shows the ancestral state reconstruction of habitat environment (PC1 and PC2 scores). (b, c) Means ± SEM of (b) PC1 and (c) PC2 scores of species in each cecum type (*n *=* *15 for manicate type, *n *= 10 for simple type)

Our phylogenetic tree indicated that the manicate cecum type evolved from the simple cecum type once, and each cecum type was clustered in the tree (Figure 9a). The PC1 score was higher and the PC2 score was lower in the lineage after the evolutionary change from simple to manicate cecum type than in the lineage with simple cecum type, according to the phylogenetic generalized least squares method (Figure 9b, c; PC1: AIC for the model with cecum evolution: 89.1, AIC for the null model: 89.7; PC2: AIC for the model with cecum evolution: 67.0, AIC for the null model: 67.5). The sites with a higher PC1 score should be suitable habitat for underground decomposers owing to sufficient habitat space with moist, soft, carbon‐rich soil, which would provide abundant food resources for earthworms (see Appendix S1). The sites with a lower PC2 score should be suitable habitat for litter decomposers owing to a deep and nutritionally rich litter layer, as a litter layer with a low C:N ratio has a high nitrogen concentration.

## 4 DISCUSSION

The Unifrac distance was positively correlated with geographic distance in the haplotype tree, and none of the haplotypes were shared among sites. These results indicate a very low rate of migration among primary forests due to the earthworms’ poor dispersal ability. However, this pattern was not detected in the MOTU tree or at the Megascolecidae node in the haplotype tree, suggesting that the low rate of migration in earthworms causes geographic isolation at a relatively short timescale. Such a trend is likely common in soil‐inhabiting animals, because their dispersal ability is considered to be poorer than that of aboveground animals (Chang et al., 2008; Minamiya et al., 2009). The local underground community assemblages have differentiated among primary forests and are unique to each area, revealing a long divergence history. Changes in the distributions of soil animal communities likely occur slowly, raising concerns about their ability to keep pace with the rapid climate change occurring due to human activity.

Our results also show that the community assembly process of megascolecid earthworms has been affected by evolutionary factors related to the habitat environment. Habitat preference changed due to the evolutionary change in the intestinal cecum. Habitat preference was clustered in the megascolecid phylogeny, indicating niche conservatism. The PC1 score was higher and the PC2 score was lower in the lineage with manicate cecum type than in the lineage with simple cecum type. The sites with higher PC1 and lower PC2 scores have suitable habitat for underground decomposers, especially for litter decomposers, with a deep and nutritionally rich litter layer, moist and soft soil, and abundant food resources. Our results indicate that earthworms with manicate intestinal ceca favor habitats in the litter layer and surroundings, where they feed on less decomposed organic matter. The adaptation to such a novel habitat due to the evolutionary change in the intestinal cecum is the driving force for habitat range expansion in the megascolecid lineage. Thus, our results show that the evolutionary change in a key trait has greatly affected the process of community assembly over an evolutionary timescale.

In contrast, our findings indicate that evolutionary factors have not affected the process of community assembly in the lumbricid lineage over an evolutionary timescale, whereas biogeographic factors have affected it (Figures 7 and 8; Tables 2 and 3). Although some lumbricid species live in the soil layer and others in the litter layer (Bouché, 1977; Domínguez et al., 2015; Perel, 1977), we detected no evolutionary pattern of habitat preference in our study. This pattern would be partly caused by low species diversity in the Lumbricidae in Japanese primary forests. In addition, the fundamental niche width in lumbricid species may be wider than that in megascolecid species.

Using earthworms as a model system, we demonstrated that the underground animal community of each area was unique and had a long divergence history affected by biogeographic factors owing to the animals’ poor dispersal ability. Although previous studies for soil animals have not investigated the evolutionary ecology of habitat adaptation, our findings also show that the process of community assembly in megascolecid earthworms has been driven by the evolution of intestinal cecum morphologies through the change of habitat preference. We found a clear difference in the community assembly process between the Megascolecidae and the Lumbricidae, even though species from both lineages live sympatrically. Our results suggest that investigating the evolution of a key trait, such as the intestinal cecum, that is closely related to life history can lead to the clear description of the community assembly process over an evolutionary timescale.

## AUTHOR CONTRIBUTIONS

H.I., K.F., M.T.I., and K.M. designed the study. H.I. and K.F. conducted fieldwork. H.I., E. S.‐K., M.T., and Y.S. conducted experiments and analyzed data. H.I wrote the first draft of the paper.
