# Supplementary material for: Evolution of a key trait greatly affects underground community assembly process through habitat adaptation in earthworms
Source: Ecol Evol. 2018 Jan 8;8(3):1726–35. doi: 10.1002/ece3.3777 (PMC5792615; doi:10.1002/ece3.3777)
Supplement: Supplementary file 1 [file ECE3-8-1726-s001.doc]

**Appendix S1** Latitude, longitude, elevation, environmental factors, and PC1 and PC2 scores at each site.

|  |  |  | |  | | | Environmental factors for litter layer | | | | | | | | | |
| --- | --- | --- | --- | --- | --- | --- | --- | --- | --- | --- | --- | --- | --- | --- | --- | --- |
| Site | Latitude | | Longitude | | Elevation | Litter depth in  summer (cm) | | | Litter depth in autumn (cm) | | Dry weight (g) | | Carbon content | | C:N ratio | |
|  | (º) | | (º) | | (m) | average | | SD | average | SD | average | SD | average | SD | average | SD |
| 1 | 35.94 | | 138.80 | | 1240 | 2.88 | | 1.32 | 3.16 | 0.48 | 29.65 | 12.91 | 0.504 | 0.003 | 35.5 | 4.14 |
| 2 | 36.94 | | 140.59 | | 650 | 3.92 | | 1.06 | 3.96 | 0.17 | 21.35 | 6.77 | 0.502 | 0.003 | 28.6 | 1.29 |
| 3 | 35.32 | | 138.73 | | 1680 | 2.96 | | 0.43 | 2.20 | 0.58 | 47.26 | 26.70 | 0.491 | 0.017 | 29.5 | 2.73 |
| 4 | 35.92 | | 138.80 | | 1800 | 2.04 | | 0.55 | 2.20 | 1.17 | 23.40 | 12.08 | 0.472 | 0.048 | 41.1 | 5.02 |
| 5 | 35.74 | | 138.21 | | 1920 | 2.24 | | 0.62 | 2.16 | 0.33 | 9.68 | 5.73 | 0.510 | 0.032 | 33.7 | 4.72 |
| 6 | 36.74 | | 137.82 | | 1070 | 3.32 | | 0.33 | 2.88 | 0.56 | 27.62 | 5.10 | 0.500 | 0.012 | 30.4 | 2.63 |
| 7 | 36.85 | | 138.08 | | 1280 | 4.84 | | 0.82 | 3.52 | 0.36 | 28.52 | 10.58 | 0.508 | 0.006 | 27.3 | 1.24 |
| 8 | 36.83 | | 138.47 | | 1280 | 4.32 | | 0.41 | 4.16 | 0.46 | 21.70 | 4.99 | 0.500 | 0.007 | 27.3 | 1.36 |
| 9 | 36.99 | | 138.40 | | 1090 | 3.68 | | 0.67 | 3.88 | 1.44 | 32.34 | 4.32 | 0.522 | 0.002 | 30.7 | 2.29 |
| 10 | 36.79 | | 139.07 | | 1290 | 4.00 | | 0.58 | 4.00 | 0.32 | 22.22 | 5.92 | 0.507 | 0.003 | 23.3 | 0.85 |
| 11 | 36.84 | | 139.17 | | 1520 | 3.28 | | 0.46 | 4.68 | 0.72 | 42.30 | 9.61 | 0.510 | 0.004 | 32.1 | 0.65 |
| 12 | 36.88 | | 139.42 | | 1330 | 3.24 | | 0.22 | 3.56 | 0.52 | 37.30 | 12.19 | 0.500 | 0.004 | 29.7 | 1.61 |

| Environmental factors for soil layer | | | |  |  |  |  |  |  |  |  |  | PC scores | |
| --- | --- | --- | --- | --- | --- | --- | --- | --- | --- | --- | --- | --- | --- | --- |
| Depth of A1 layer (cm) | | Bulk density (g) | | Water content | | pH | | Carbon content | | C:N ratio | |  |  |  |
| average | SD | average | SD | average | SD | average | SD | average | SD | average | SD |  | PC1 | PC2 |
| 1.60 | 0.65 | 121.7 | 23.9 | 0.64 | 0.049 | 4.19 | 0.157 | 0.19 | 0.059 | 14.9 | 0.65 |  | -1.795 | 0.090 |
| 3.10 | 0.55 | 172.7 | 32.6 | 0.48 | 0.042 | 4.40 | 0.189 | 0.14 | 0.035 | 14.5 | 0.50 |  | -2.345 | -2.362 |
| 4.33 | 1.15 | 88.3 | 33.5 | 0.65 | 0.051 | 4.83 | 0.278 | 0.27 | 0.066 | 14.6 | 0.83 |  | -0.894 | 0.142 |
| 4.38 | 1.38 | 88.5 | 26.5 | 0.60 | 0.079 | 3.59 | 0.088 | 0.26 | 0.098 | 19.0 | 2.52 |  | -1.273 | 4.142 |
| 4.00 | 1.00 | 147.8 | 77.2 | 0.43 | 0.119 | 4.11 | 0.473 | 0.12 | 0.048 | 17.1 | 2.86 |  | -3.338 | 0.297 |
| 4.67 | 1.53 | 94.5 | 30.5 | 0.69 | 0.042 | 3.90 | 0.216 | 0.26 | 0.094 | 19.0 | 1.68 |  | 0.504 | 1.237 |
| 6.33 | 1.15 | 52.0 | 6.2 | 0.79 | 0.024 | 3.68 | 0.214 | 0.41 | 0.018 | 18.1 | 0.99 |  | 3.526 | 0.452 |
| 7.33 | 0.58 | 108.3 | 70.5 | 0.71 | 0.115 | 3.88 | 0.503 | 0.26 | 0.121 | 16.3 | 1.08 |  | 1.522 | -1.020 |
| 5.33 | 0.58 | 119.6 | 5.6 | 0.66 | 0.015 | 3.64 | 0.140 | 0.22 | 0.012 | 16.4 | 0.43 |  | 0.722 | -0.933 |
| 7.00 | 1.00 | 98.1 | 11.2 | 0.64 | 0.016 | 3.82 | 0.059 | 0.26 | 0.019 | 16.6 | 0.81 |  | 1.401 | -1.266 |
| 7.00 | 0.00 | 69.3 | 4.0 | 0.77 | 0.006 | 3.58 | 0.076 | 0.33 | 0.017 | 17.8 | 0.61 |  | 2.828 | 0.578 |
| 7.33 | 1.15 | 139.4 | 16.7 | 0.63 | 0.047 | 4.59 | 0.155 | 0.13 | 0.023 | 15.7 | 0.67 |  | -0.856 | -1.357 |

**Appendix S2** Mean number of collected earthworms per quadrat at each site.

| Site | Season | Sampling date | The number of collected earthworms per quadrat | | | | | | | | | | | | | |
| --- | --- | --- | --- | --- | --- | --- | --- | --- | --- | --- | --- | --- | --- | --- | --- | --- |
| No. |  |  | Megascolecidae | | | |  | Lumbricidae | | | |  | Moniligastridae | | | |
|  |  |  | Litter layer | | Soil layer | |  | Litter layer | | Soil layer | |  | Litter layer | | Soil layer | |
|  |  |  | average | SD | average | SD |  | average | SD | average | SD |  | average | SD | average | SD |
| 1 | summer | July 4, 2010 | 0.6 | 0.55 | 2.0 | 2.45 |  | 0.0 | 0.00 | 1.4 | 1.14 |  | 0.0 | 0.00 | 0.0 | 0.00 |
|  | autumn | Aug. 26, 2010 | 0.2 | 0.45 | 1.4 | 1.14 |  | 0.0 | 0.00 | 3.0 | 2.35 |  | 0.0 | 0.00 | 0.0 | 0.00 |
| 2 | summer | July 6, 2010 | 3.2 | 2.39 | 1.6 | 1.14 |  | 0.8 | 1.79 | 1.4 | 1.95 |  | 0.0 | 0.00 | 0.0 | 0.00 |
|  | autumn | Aug. 24, 2010 | 1.6 | 1.52 | 0.6 | 0.89 |  | 0.0 | 0.00 | 2.8 | 1.92 |  | 0.0 | 0.00 | 0.0 | 0.00 |
| 3 | summer | July 1, 2012 | 0.8 | 1.30 | 1.6 | 1.14 |  | 0.0 | 0.00 | 1.4 | 0.89 |  | 0.0 | 0.00 | 0.0 | 0.00 |
|  | autumn | Aug. 30, 2012 | 2.8 | 2.77 | 16.6 | 8.26 |  | 0.2 | 0.45 | 4.2 | 2.28 |  | 0.0 | 0.00 | 0.0 | 0.00 |
| 4 | summer | July 2, 2012 | 0.0 | 0.00 | 0.0 | 0.00 |  | 0.4 | 0.89 | 2.4 | 1.67 |  | 0.0 | 0.00 | 0.0 | 0.00 |
|  | autumn | Aug. 31, 2012 | 0.0 | 0.00 | 1.0 | 1.00 |  | 0.0 | 0.00 | 3.4 | 2.88 |  | 0.0 | 0.00 | 0.0 | 0.00 |
| 5 | summer | July 3, 2012 | 0.0 | 0.00 | 3.0 | 3.54 |  | 0.2 | 0.45 | 1.6 | 1.82 |  | 0.0 | 0.00 | 0.0 | 0.00 |
|  | autumn | Sep. 1, 2012 | 0.0 | 0.00 | 4.8 | 5.26 |  | 0.0 | 0.00 | 0.2 | 0.45 |  | 0.0 | 0.00 | 0.0 | 0.00 |
| 6 | summer | July 5, 2012 | 0.0 | 0.00 | 0.0 | 0.00 |  | 0.0 | 0.00 | 2.8 | 2.77 |  | 0.0 | 0.00 | 3.8 | 3.56 |
|  | autumn | Sep. 2, 2012 | 0.0 | 0.00 | 0.0 | 0.00 |  | 0.0 | 0.00 | 1.8 | 2.68 |  | 0.0 | 0.00 | 4.4 | 3.21 |
| 7 | summer | July 6, 2012 | 0.0 | 0.00 | 0.0 | 0.00 |  | 0.2 | 0.45 | 3.2 | 6.61 |  | 0.0 | 0.00 | 0.0 | 0.00 |
|  | autumn | Sep. 3, 2012 | 0.0 | 0.00 | 0.0 | 0.00 |  | 0.4 | 0.89 | 1.8 | 1.92 |  | 0.0 | 0.00 | 0.0 | 0.00 |
| 8 | summer | July 7 and 8, 2012 | 1.0 | 1.22 | 0.8 | 0.84 |  | 0.0 | 0.00 | 0.8 | 1.30 |  | 0.0 | 0.00 | 0.0 | 0.00 |
|  | autumn | Sep. 4, 2012 | 0.6 | 0.89 | 0.0 | 0.00 |  | 0.4 | 0.55 | 2.0 | 3.08 |  | 0.0 | 0.00 | 0.0 | 0.00 |
| 9 | summer | July 9, 2012 | 0.0 | 0.00 | 0.0 | 0.00 |  | 0.0 | 0.00 | 0.8 | 1.10 |  | 0.0 | 0.00 | 0.0 | 0.00 |
|  | autumn | Sep. 5, 2012 | 0.0 | 0.00 | 0.0 | 0.00 |  | 0.0 | 0.00 | 0.0 | 0.00 |  | 0.0 | 0.00 | 0.0 | 0.00 |
| 10 | summer | July 11 and 12, 2012 | 0.4 | 0.55 | 0.0 | 0.00 |  | 0.2 | 0.45 | 2.0 | 2.24 |  | 0.0 | 0.00 | 0.0 | 0.00 |
|  | autumn | Sep. 6, 2012 | 0.2 | 0.45 | 0.0 | 0.00 |  | 0.2 | 0.45 | 2.0 | 1.58 |  | 0.0 | 0.00 | 0.0 | 0.00 |
| 11 | summer | July 13, 2012 | 0.0 | 0.00 | 0.0 | 0.00 |  | 0.0 | 0.00 | 3.8 | 4.49 |  | 0.0 | 0.00 | 0.0 | 0.00 |
|  | autumn | Sep. 7, 2012 | 0.0 | 0.00 | 0.0 | 0.00 |  | 0.0 | 0.00 | 3.8 | 2.59 |  | 0.0 | 0.00 | 0.0 | 0.00 |
| 12 | summer | July 14, 2012 | 0.8 | 0.45 | 0.2 | 0.45 |  | 0.2 | 0.45 | 3.4 | 3.65 |  | 0.0 | 0.00 | 0.0 | 0.00 |
|  | autumn | Sep. 8, 2012 | 0.2 | 0.45 | 0.0 | 0.00 |  | 0.4 | 0.89 | 2.8 | 3.03 |  | 0.0 | 0.00 | 0.0 | 0.00 |
